# Impact of insight quality on treatment adherence in schizophrenia

**DOI:** 10.1192/j.eurpsy.2023.579

**Published:** 2023-07-19

**Authors:** A. Rami, E. Sana, C. Mejda, D. Rahma

**Affiliations:** ^1^Ibn Omrane; ^2^Manouba_Tunisia, Razi Hospital, Manouba, Tunisia

## Abstract

**Introduction:**

Schizophrenia is a chronic, frequent, and disabling psychiatric condition. The prognosis is more severe in the absence of treatment.

**Objectives:**

The aims of our study were to evaluate the quality of treatment adherence and the quality of insight of patients with schizophrenia and to assess the implication of these factors as predictors of poor adherence.

**Methods:**

We conducted a cross-sectional and analytical study. We recruited 150 patients with schizophrenia treated at Razi Hospital of Manouba, divided into 113 patients with good adherence compared to 37 patients with poor adherence. We used the Medical Adherence Report Scale (MARS) to assess the quality of therapeutic adherence and the Birchwood Insight Scale for Insight Assessment.

**Results:**

Poor treatment adherence in patients with schizophrenia was significantly associated with poor insight (p=0.001). Good adherence was associated with positive perception of treatment effectiveness (p<0.001). The predictive factor for poor adherence to therapy in multivariate analysis, after adjusting for the confounding variables was the negative perception of side effects (p=0.02). The predictive factor for good adherence was the presence of insight into the need for treatment (p=0.002).

**Conclusions:**

To prevent poor treatment adherence, a systematic screening for predictive factors and adequate management of schizophrenia would be imperative.

**Disclosure of Interest:**

A. Rami Shareolder of: no, Grant / Research support from: no, Consultant of: no, Employee of: no, Paid Instructor of: no, Speakers bureau of: no, E. Sana Shareolder of: no, Grant / Research support from: no, Consultant of: no, Employee of: no, Paid Instructor of: no, Speakers bureau of: no, C. Mejda Shareolder of: no, Grant / Research support from: no, Consultant of: no, Employee of: no, Paid Instructor of: no, Speakers bureau of: no, D. Rahma Shareolder of: no, Grant / Research support from: no, Consultant of: no, Employee of: no, Paid Instructor of: no, Speakers bureau of: no

